# The Beavis Effect in Next-Generation Mapping Panels in *Drosophila melanogaster*

**DOI:** 10.1534/g3.117.041426

**Published:** 2017-06-05

**Authors:** Elizabeth G. King, Anthony D. Long

**Affiliations:** * Division of Biological Sciences, University of Missouri, Columbia, Missouri 65211; † Department of Ecology and Evolutionary Biology, University of California Irvine, California 92697

**Keywords:** complex traits, *Drosophila melanogaster*, Beavis effect, QTL mapping, GWAS, DSPR, DGRP, multiparental populations, MPP

## Abstract

A major goal in the analysis of complex traits is to partition the observed genetic variation in a trait into components due to individual loci and perhaps variants within those loci. However, in both QTL mapping and genetic association studies, the estimated percent variation attributable to a QTL is upwardly biased conditional on it being discovered. This bias was first described in two-way QTL mapping experiments by William Beavis, and has been referred to extensively as “the Beavis effect.” The Beavis effect is likely to occur in multiparent population (MPP) panels as well as collections of sequenced lines used for genome-wide association studies (GWAS). However, the strength of the Beavis effect is unknown—and often implicitly assumed to be negligible—when “hits” are obtained from an association panel consisting of hundreds of inbred lines tested across millions of SNPs, or in multiparent mapping populations where mapping involves fitting a complex statistical model with several d.f. at thousands of genetic intervals. To estimate the size of the effect in more complex panels, we performed simulations of both biallelic and multiallelic QTL in two major *Drosophila melanogaster* mapping panels, the GWAS-based *Drosophila* Genetic Reference Panel (DGRP), and the MPP the *Drosophila* Synthetic Population Resource (DSPR). Our results show that overestimation is determined most strongly by sample size and is only minimally impacted by the mapping design. When < 100, 200, 500, and 1000 lines are employed, the variance attributable to hits is inflated by factors of 6, 3, 1.5, and 1.1, respectively, for a QTL that truly contributes 5% to the variation in the trait. This overestimation indicates that QTL could be difficult to validate in follow-up replication experiments where additional individuals are examined. Further, QTL could be difficult to cross-validate between the two *Drosophila* resources. We provide guidelines for: (1) the sample sizes necessary to accurately estimate the percent variance to an identified QTL, (2) the conditions under which one is likely to replicate a mapped QTL in a second study using the same mapping population, and (3) the conditions under which a QTL mapped in one mapping panel is likely to replicate in the other (DGRP and DSPR).

One of the most fundamental questions about the genetic basis of complex traits is: how much does each individual locus contribute to the total genetic variance that exists for a phenotype (Falconer and Mackay 1996; Roff 1997; Lynch and Walsh 1998)? Are there few loci contributing substantial amounts or many small loci, each with marginal contributions? Obviously, answering these questions requires the ability to estimate the magnitude of the contribution of a locus (*i.e.*, QTL) to a phenotype. However, this straightforward goal is complicated by the Beavis effect [first described in Beavis *et al.* (1991) and Beavis (1994)], the well-known phenomenon that when the percentage of variance explained (hereafter PVE) by a locus is estimated only for statistically significant loci, the PVE will be overestimated on average, and in some cases severely so. Both Utz and Melchinger (1994) and Beavis (1998) performed simulations demonstrating this bias for classic two-line cross designs. They demonstrated that the severity of the bias increases with decreasing power, thus the bias is expected to be greatest when sample size is low and/or when the true contribution of a locus is small. Xu (2003) analytically derived an equation predicting the severity of the bias for a backcross QTL mapping design, which agreed with the original simulation results of Beavis (1998). Thus, the statistical properties of the Beavis effect have been well characterized for traditional two-line QTL mapping designs; however, it is not known how precisely these results apply to modern mapping approaches.

Modern, “next-generation” mapping panels differ in several key ways that could influence the statistical properties of the Beavis effect in those panels. Here, we focus on GWAS panels and MPPs. GWAS perform single marker association tests from sets of individuals or lines, associating the genotype at a locus with the phenotype. The bias associated with GWAS has been noted previously (Göring *et al.* 2001; Hirschhorn and Daly 2005) in human studies. In model system GWAS, tests for association can be carried out at millions of SNPs in panels of a few hundred inbred lines (*e.g.*, Flint-Garcia *et al.* 2005; Mackay *et al.* 2012; Alonso-Blanco *et al.* 2016). MPPs are more similar to traditional QTL mapping designs, but involve a multi-way initial cross between founder strains followed by multiple generations of intercrossing (Kover *et al.* 2009; Aylor *et al.* 2011; King *et al.* 2012a,b; Cubillos *et al.* 2013; Long *et al.* 2014). In these populations, mapping involves associating the haplotype at a locus with the phenotype of interest. The number of possible haplotypes at a locus is equal to the number of contributing parents in the MPP, leading to more complex models with more d.f. than the single degree of freedom test typically carried out in a genetic association study. One major difference in modern panels is the frequency distribution of alleles in these panels. The frequency of the alleles at a causative locus is known to affect the power to detect it, with maximal power when alleles are at 0.5 and reduced power as one allele becomes rare (Slager *et al.* 2000). In traditional two-line intercross designs, allele frequencies rarely deviate much from a frequency of 0.5. However, the expected frequency distributions for GWAS panels and MPP panels are quite different (*e.g.*, Kover *et al.* 2009; Aylor *et al.* 2011; King *et al.* 2012a). In GWAS-based panels, alleles are at their natural allele frequencies and thus, there are many alleles at low frequency. In MPPs, alleles are expected to be at a frequency of 1/*n*, where n equals the number of parents. However, particularly when there is a longer crossing period, drift and selection will cause true frequencies to deviate, in some cases substantially, from expectation (King *et al.* 2012a; Chesler *et al.* 2016). In addition, modern mapping panels have higher marker density, and thus less missing genotype information. In both types of panels, genome information is increasingly approaching complete genome information, with full resequencing of all lines in GWAS panels and full resequencing of parent lines coupled with dense genotyping of recombinant inbred lines (RILs) in MPPs. All of the above factors have the potential to influence the degree of bias in estimating the contribution of individual QTL to the genetic variance.

When the Beavis effect is substantial, there is not an expectation of high concordance among different mapping studies. One would not necessarily expect to map the same QTL in replicated experiments or in different mapping panels. Further, for successfully mapped QTL, one might expect estimated effect sizes to differ, perhaps considerably. In the past, a failure to appreciate this reality has led to results that are consistent with one another being incorrectly described as contradictory (*e.g.*, Altshuler *et al.* 2000). In addition, in some cases, complex hypotheses are invoked to explain inconsistent results for the same loci that could also be explained by the Beavis effect (*e.g.*, Huang *et al.* 2012). Therefore, it is valuable to clarify the expectations for the validation of mapped QTL within the same mapping populations and between different mapping populations.

In this paper, we focus on two next-generation mapping panels used widely in the *Drosophila melanogaster* community, the GWAS-based DGRP and the MPP DSPR. We simulate QTL that account for different true PVE and map these simulated QTL for different sample sizes in both panels. We then quantify the resulting bias in the estimates of the PVE by significant QTL. Finally, we discuss the factors that influence the likelihood of replicating a QTL in a second mapping study of the same design, as well as the ability to replicate mapped factors between resources.

## Methods

### Mapping populations

We used two major community resources for genetic mapping in the *D. melanogaster* system, the DGRP (http://dgrp2.gnets.ncsu.edu/data.html; Mackay *et al.* 2012; Huang *et al.* 2014) and the DSPR (http://FlyRILs.org; King *et al.* 2012a,b), to perform simulations. All simulations and analyses described below were performed in R (version 3.3.1; R Core Team 2016) and all code is publicly available via GitHub (https://github.com/egking/QTLbiasSIM). The same set of code with nearly all raw and intermediate data files is available at Zenodo: http://doi.org/10.5281/zenodo.438140.

For the DGRP, we used the DGRP Freeze 2.0 release, consisting of 205 inbred lines (http://dgrp2.gnets.ncsu.edu/data.html) created from wild-caught females in Raleigh, NC. All lines have been resequenced, and genetic variants have been called. Details about the formation and sequencing of this mapping population are available in Mackay *et al.* (2012) and Huang *et al.* (2014). Briefly, the DGRP lines were created by collecting over 1000 mated females and establishing isofemale lines, which were then subjected to 20 generations of full-sib mating. To quantify genetic similarity between lines, we used the A.mat function in the rrBLUP package to obtain a global kinship matrix (Endelman 2011; Poland *et al.* 2012). We included only positions with a call in 80% of lines and a minor allele frequency > 5%. Subsequently, we dropped one member of each set of lines whose coefficient of coancestry exceeded 0.25. A total of 20 lines were dropped, giving a final set of 185 lines.

For the DSPR, we used the data from the DSPR release 4 (http://FlyRILs.org/Data), which consists of two populations (pA and pB) of RILs, each created from an eight-way, 50 generation cross. Following the crossing phase, lines were subjected to 20–25 generations of full-sib mating. The two populations of RILs are essentially replicates; each was created from a different set of eight founder lines with the exception of a single founder line that is shared between the two populations. The founder lines have been fully resequenced and all RILs have been genotyped using RAD markers. These data inform a hidden Markov model (HMM) that infers the probability that each segment in each RIL is derived from each founder line, producing near complete genome information for all RILs (King *et al.* 2012b). All data associated with the DSPR lines are available here: http://FlyRILs.org/Data. Complete details about the formation and genetic characterization of the DSPR are available in King *et al.* (2012a,b). For our simulations below, we used only the pA RILs and expect that the results would apply equally to pB RILs.

### Simulated phenotypes

For the DSPR, we used the set of HMM probabilities (King *et al.* 2012b) and founder genotype calls to impute full genotype data for each RIL corresponding to the probability of a given SNP flavor at each location for each RIL. For a given RIL at a given position, the probability it harbors the reference allele is given by:

$$\mathrm{Prob}\left(\mathrm{REF}\right)=\sum_{i=1}^{8}F_{i}\cdot P_{i},$$

where *F_i_* is the genotype for the *i*th founder (0 = alternative allele, 1 = reference allele) and *P_i_* is the probability the RIL harbors the *i*th founder genotype at the position. We then dropped any positions with a minor allele frequency < 2.5%. We also excluded positions that were outside the set of genotyped positions in the RILs, where we therefore do not have a haplotype assignment from our HMM. At each position where we simulated a QTL, we used the binomial distribution to assign either the reference or alternative allele to each RIL using the probabilities calculated above. Since founder allelic states are inferred with near 100% accuracy for most loci (*i.e.*, the *F_i_*’s), and similarly at most positions in the genome in any given RIL the founder state is also highly certain (*i.e.*, the *P_i_*’s), for any given RIL at any given position the Prob(REF) is close to zero or one. In fact, over all RILs and SNPs only 4% of Prob(REF) inferences are between 2.5 and 97.5%. We simulated sample sizes of 100, 185 (to match the DGRP), 500, and 878 (the size of the full pA panel).

In the DGRP, we dropped any positions with a minor allele frequency < 2.5% in the resource or that was missing from > 20% of lines. We then used the kinship-based EM method implemented in the A.mat function in the rrBLUP package to impute any missing genotypes. In addition, when selecting sites to simulate QTL, we excluded positions that were near major inversions (Release five coordinates = *2L:* 0.4–14.9 Mb, *2R*: 9–18 Mb, and *3R*: 6–27 Mb). We considered sample sizes of 100 and 185 (the size of the full DGRP panel).

We then simulated both biallelic and multiallelic QTL for different values of the PVE by the QTL (5, 10, and 20%) in both mapping populations. In the biallelic case, we simulated a QTL at a given position by randomly choosing a SNP and adding environmental variance to correspond to the given PVE by the QTL to generate a phenotype. We generated a set of random normal deviates $N\left\{\mathrm{\mu =0,\sigma}=\sqrt{\left[\left(1-z\right)/z\right]\cdot \sigma_{G}^{2}}\right\}$ to correspond to environmental variance for each effect size where *z* = the percent of the phenotypic variance explained by the QTL and $\sigma_{G}^{2}$ is the genetic variance at the QTL. We also simulated a gene-based multiallelic case (c.f. Thornton *et al.* 2013; Long *et al.* 2014). For each gene, we chose three random SNPs within the interval of that gene ± 1 kb on either side. We then assigned a multiallelic additive genotype for each line by summing across the three SNPs (*e.g.*, 000 = 0, 001 = 1, 011 = 2, 010 = 1… 0.111 = 3). We then added environmental variance as above to create phenotypes. In most cases, we performed 1000 independent simulations, simulating a single QTL in each at randomly chosen loci. However, when power is low, few QTL are detected, producing a small sample size to estimate the average PVE. Therefore, to ensure a reasonable estimate of the PVE by detected QTL, we performed additional simulations whenever the number detected was < 30 (the number of simulations is given in Table 1).

**Table 1:** Power (% QTL identified) and % validated in the DGRP and DSPR for different sample sizes and true PVE by the simulated QTL

		DGRP						DSPR					
		Biallelic			Multiallelic			Biallelic			Multiallelic		
True PVE	Sample Size	*N**^a^*	Mapped*^b^*	% Validated*^c^*	*N**^a^*	Mapped*^b^*	% Validated*^c^*	*N**^a^*	Mapped (%)*^b^*	% Validated*^c^*	*N**^a^*	Mapped (%)*^b^*	% Validated*^c^*
5	100	6000	38 (0.6%)	13.1%	6000	35 (0.6%)	2.9%	2000	25 (1.3)	16.0	2000	24 (1.2)	8.3
5	185	3000	49 (1.6%)	4.1%	5000	51 (1.0%)	5.9%	1000	69 (6.9)	17.4	1000	26 (4.6)	45.6
10	100	3000	50 (1.6%)	6.0%	3000	38 (1.3%)	5.3%	1000	80 (8.0)	33.7	1000	62 (6.2)	33.9
10	185	1000	149 (14.9%)	31.0%	1000	56 (5.6%)	33.9%	1000	304 (30.4)	73.0	1000	309 (30.9)	63.0
20	100	1000	216 (21.6%)	46.0%	1000	87 (8.7%)	34.5%	1000	388 (38.8)	74.0	1000	373 (37.3)	68.0
20	185	1000	852 (85.2%)	93.0%	1000	456 (45.6%)	75.0%	1000	876 (87.6)	97.0	1000	872 (87.2)	98.0
5	500	—	—	—	—	—	—	1000	482 (48.2)	90.0	1000	539 (53.9)	83.0
5	878	—	—	—	—	—	—	1000	928 (92.8)	99.0	1000	929 (92.9)	97.0
10	500	—	—	—	—	—	—	1000	967 (96.7)	100	1000	967 (96.7)	99.0
10	878	—	—	—	—	—	—	1000	996 (99.6)	100	1000	994 (99.4)	100
20	500	—	—	—	—	—	—	1000	997 (99.7)	100	1000	993 (99.3)	100
20	878	—	—	—	—	—	—	1000	997 (99.7)	100	1000	996 (99.6)	100

DGRP, *Drosophila* Genetic Reference Panel; DSRP, *Drosophila* Synthetic Population Resource; PVE, percentage of variance explained; QTL, quantitative trait loci.

a The number of simulations performed. Note that we simulated an increased number when power was low to generate enough mapped QTL to estimate the observed PVE accurately.

b The number of simulations resulting in a significant, mapped QTL. The percentage (*i.e.*, power) is in parentheses.

c The percentage of the mapped QTL that were identified in a second set of simulated QTL within the population (see *Methods* for details).

In both the DSPR and DGRP, the fact that all individuals of a given line are genetically identical allows for multiple measures per line to reduce environmental error, effectively increasing the PVE by the QTL. All our simulations only considered a single QTL, thus *V*_e_ should be thought of as a measure of the true random environmental noise plus the genetic variance due to all QTL in gametic phase linkage equilibrium with the focus locus. In both the DSPR and DGRP, linkage disequilibrium in the ILs/RILs only extends over small genetic intervals, so for major QTL this is a reasonable assumption. In the DGRP, linkage disequilibrium decays rapidly, reaching background levels within hundreds of base pairs (Mackay *et al.* 2012). In the DSPR, linkage extends further but decays to background within ∼2 Mb (Figure S2 in File S1).

### Mapping simulated QTL

When performing genetic mapping, it is necessary to account for relatedness between lines, particularly when genetic similarity varies substantially between different sets of lines. However, including the causative marker in the calculation of the kinship matrix is known to produce overly conservative mapping results (Yang *et al.* 2014), which we confirmed with our simulations (Figure S1 in File S1). To avoid this effect, we used the leave-one-chromosome-out (LOCO) method and computed separate kinship matrices leaving one chromosome arm out at a time (Yang *et al.* 2014). When testing a given marker, we used the corresponding kinship matrix that does not include that chromosome arm. Other approaches to mapping that could be implemented to avoid overly conservative results are the NAM-R (Xavier *et al.* 2015) R package or the BayesCpi function in the gdmp (Abdel-Azim 2016) R package. For the DGRP, we used the A.mat function in the rrBLUP package to obtain these kinship matrices (Endelman 2011; Poland *et al.* 2012). For the DSPR, we estimated the kinship matrix using the haplotype inferences stemming from the HMM as the proportion of the genome identical-by-descent between any pair of RILs, using ∼11,000 regularly spaced positions on a genetic scale. For any given pair of RILs (a and b), the proportion of the genome identical by descent is given by,

$$\frac{\sum_{j=1}^{n}\sum_{i=1}^{8}F_{i,j,a}\cdot F_{i,j,b}}{n}$$

where $F_{i,j,a}$ is the probability the *a*th RIL harbors the *i*th founder genotype at the *j*th position, $F_{i,j,b}$ is the probability the *b*th RIL harbors the *i*th founder genotype at the *j*th position, and *n* is the number of positions. The DGRP consists of lines derived from a wild-caught population. Thus, haplotype blocks are short and haplotype information is not estimated. The typical model fit is a biallelic association test at each marker. We adapted the assoc.map function in the DOQTL package (Gatti *et al.* 2014) to the DGRP data to perform a linear mixed model. This function relies on the methods developed for the QTLRel package (Cheng *et al.* 2011) and the regress package (Clifford and McCullagh 2006) to account for kinship (using LOCO kinship matrices) and the Matrix eQTL algorithm for computational efficiency. The model at a given locus is as follows:

$$y_{i}=x_{i}b_{i}+g_{i}+e_{i},$$

where *y_i_* is the phenotype of the *i*th line, *x_i_* is the genotype of the *i*th line (0 = aa, 2 = AA), *b_i_* is the vector of SNP effects, *g_i_* is the vector of genetic effects to adjust for kinship, and *e_i_* is the vector of residuals. For each simulated QTL, we fitted this model at each variant within a 10 Mb (± 5 Mb) region centered on the causative SNP. We performed this localized mapping instead of genome-wide scans to reduce the computational effort involved in performing the mapping. Because we simulated a single QTL for each simulation and are only interested in the estimated PVE and significance at that QTL, we can perform mapping for only this small region.

In the DSPR, the test is for association between the founder haplotype at a given position and the phenotype (Broman and Sen 2009). We adapted the fast.qtlrel function in the DOQTL package (Gatti *et al.* 2014) to the DSPR data to fit a linear mixed model, which relies on the methods developed for the QTLRel package (Cheng *et al.* 2011) to account for kinship (using LOCO kinship matrices). The model at a given locus is as follows:

$$y_{i}=\sum_{j=1}^{7}p_{ij}b_{ij}+g_{i}+e_{i},$$

where *y_i_* is the phenotype of the *i*th individual, *p_ij_* is the probability the *i*th line has the *j*th haplotype at the locus, *b_ij_* is the vector of effects for the *j*th haplotype, *g_i_* is the vector of genetic effects to adjust for kinship, and *e_i_* is the vector of residuals. For each simulated QTL, we fitted this model at regularly spaced 10 kb intervals [see King *et al.* (2012a,b)] for a 10 Mb region (± 5 Mb of the nearest position to the causative SNP).

Performing thousands to millions of tests across the genome results in a requirement to adjust the threshold for significance to correct for performing multiple comparisons. Traditionally, QTL studies have controlled the family-wise error rate (FWER), the probability of one or more false positives occurring genome-wide, at 5%. The threshold corresponding to a 5% FWER is calculated by performing permutations and determining the maximum genome-wide LOD score for each permuted dataset. The FWER corresponding to 5% is the threshold at which there is only a 5% chance of observing at least one false positive (Churchill and Doerge 1994). Controlling the false discovery rate (FDR) (the number of false positives/the number of total positives) is also a popular approach. However, several studies (Chen and Storey 2006; Siegmund *et al.* 2011; Brzyski *et al.* 2017) have demonstrated that underlying linkage structure can make applying FDR to genome-wide tests problematic. Linkage is expected to produce similar signals across several positions, such that a single signal is expected to produce several significant “hits” nearby for both true and false positives. This dependency can alter the estimation of FDR. If positive signals can be characterized into independent signals, permutations can inform the false positive rate. We use the false positive rate here, not the FDR, as the estimate of FDR is dependent on the number of positives detected in a given genome scan. We performed 2000 genome scans for randomly generated phenotypes with no simulated QTL for each sample size. We used these scans to estimate both the threshold corresponding to a FWER of 5% and the expected number of false positives for a range of significance thresholds. To estimate the number of false positives, we first identified all peak positions for a given genome scan. Then we removed any peaks that were nearby a higher peak. We used different distances for the DSPR and DGRP given the two populations have very different linkage disequilibirum structure. For the DSPR, if a peak was within 2 cM of a higher peak, it was eliminated. For the DGRP, the same was true if a peak was within 0.5 cM. We then recalculated the Beavis effect for different *P*-value thresholds to determine how the Beavis effect relates to power and the expected false positive rate.

We considered a QTL successfully mapped if a LOD score within the 10 Mb interval surrounding the causative site exceeded the significance threshold. We estimated the PVE by the QTL from the LOD score: 1−10^−(2/^*^n^*^)*LOD^ (Broman and Sen 2009) at the peak position in this interval.

### QTL validation

We performed additional simulations to consider the likelihood of validating the significant QTL for each parameter combination described above within mapping populations. For parameter combinations where we initially identified significant QTL in over 100 simulations, we randomly selected only 100 to map again; for all others, we selected all significant QTL. We generated a new set of phenotypes for the same PVE and sample size, and performed mapping again as described above within the same mapping population.

We also performed simulations to assess the likelihood of validating a hit in a second mapping population (*i.e.*, validating a DSPR hit in the DGRP or vice versa). We limited these simulations to a set of our parameter combinations and only considered the biallelic case. For mapped QTL in the DGRP, we considered a sample size of 185 and a PVE of 5 and 10%. All significant QTL for those parameter combinations were then simulated in the DSPR for a sample size of 185 and 878. In the DSPR, we considered mapped QTL at a sample size of 185 and 878 and a PVE of 5 and 10%. These QTL were then simulated in the DGRP at a sample size of 185. We simulated QTL as above but with the effect of the locus set by the initial mapping population. Thus, at each SNP, we generated a set of random normal deviates $N\left\{\mathrm{\mu =0,\sigma}=\sqrt{\left[\left(1-z\right)/z\right]\cdot \sigma_{G}^{2}}\right\}$ to correspond to environmental variance for each effect size where *z* = the percent of the phenotypic variance explained by the QTL in the initial mapping population and $\sigma_{G}^{2}$ is the genetic variance at the QTL in the initial mapping population. This method simulates QTL in the second mapping population with a different PVE whenever the allele frequency is different. For example, a QTL with a 5% PVE in the DGRP will be simulated in the DSPR with a higher PVE if the minor allele frequency at that SNP is higher in the DSPR and vice versa.

When validating QTL, the search space is localized instead of genome-wide. Therefore, we calculated the threshold corresponding to a FWER of 5% when mapping is done only for a small region. Using our randomly generated phenotypes with no QTL, we calculated the FWER threshold by randomly choosing a position and considering only a ± 5 Mb region surrounding that position. We then used this threshold to determine the likelihood of validating a given QTL.

### Data availability

All simulations and analyses described below were performed in R (version 3.3.1; R Core Team 2016) and all code is publically available via GitHub (https://github.com/egking/QTLbiasSIM). The same set of code with nearly all raw and intermediate data files is available at Zenodo: http://doi.org/10.5281/zenodo.438140.

## Results and Discussion

### Estimating the percentage variance due to a QTL in the DGRP and DSPR

**Figure 1:**
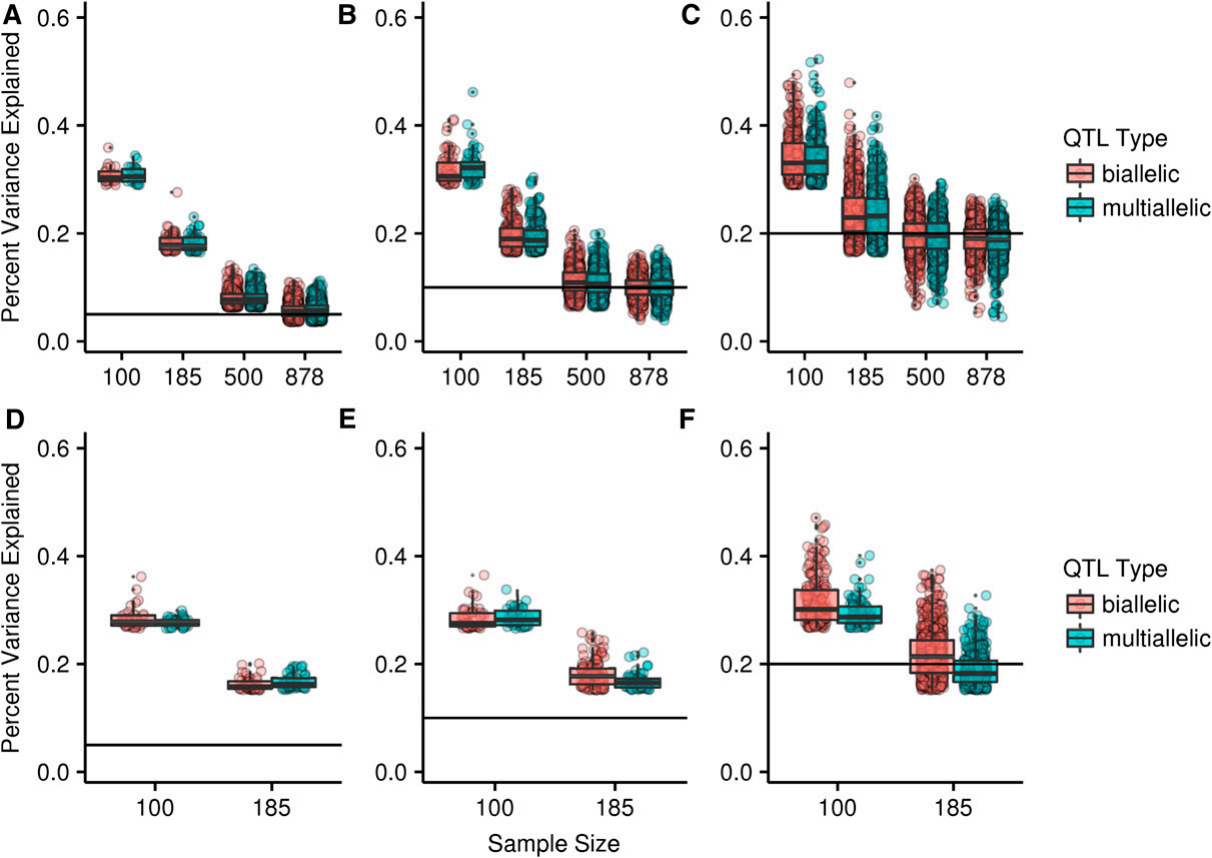
The percent variance explained by significant QTL in the DSPR (A–C) and the DGRP (D–F) for different sample sizes. The true percent variance explained by the QTL is shown by the solid line [(A and D) = 0.05, (B and E) = 0.1, and (C and F) = 0.2]. Each point corresponds to a single significant QTL. Note that different parameter combinations lead to different numbers of detected QTL and that lower powered conditions result in fewer observations. Boxplots are overlayed with the central line corresponding to the median and the box encompassing the first and third quartiles. DGRP, *Drosophila* Genetic Reference Panel; DSRP, *Drosophila* Synthetic Population Resource; QTL, quantitative trait loci.

As has been shown previously (Utz and Melchinger 1994; Beavis 1998; Xu 2003; Broman and Sen 2009), the PVE by significant QTL is overestimated in all except the most highly powered cases and the overestimation is most severe for the lowest sample sizes in both the DGRP and the DSPR (Figure 1). Power is lower in the DGRP compared to the DSPR (Table 1), resulting from a more stringent *P*-value threshold due to the fact that many more tests are performed in the DGRP, both in actual tests performed (∼11,000 *vs.* ∼2.4 million) and in the functional number of independent tests based on the size of haplotype blocks and the extent of linkage across the genome. However, the thresholds corresponding to the PVE are similar between the two populations, leading to similar overall magnitudes of the Beavis effect.

In the DSPR, where the haplotype encompasses the complete multiallelic effect, there is little difference between the biallelic and multiallelic cases. In contrast, in the DGRP, each marker is tested separately. Therefore, the actual contribution of individual SNPs is lower than the overall effect at the QTL. Thus, in the multiallelic case in the DGRP, the overestimation, when it occurs, is even greater than it appears. In the highest powered case, the multiallelic case underestimates the effect at the QTL as a whole, because it is estimated from only a single contributing SNP. Association tests that attempt to consider all SNPs within a given gene, such as burden tests, may better estimate the contribution of multiple SNPs; however, several possible methods exist that vary in their power under different genetic architectures (Thornton *et al.* 2013).

**Figure 2:**
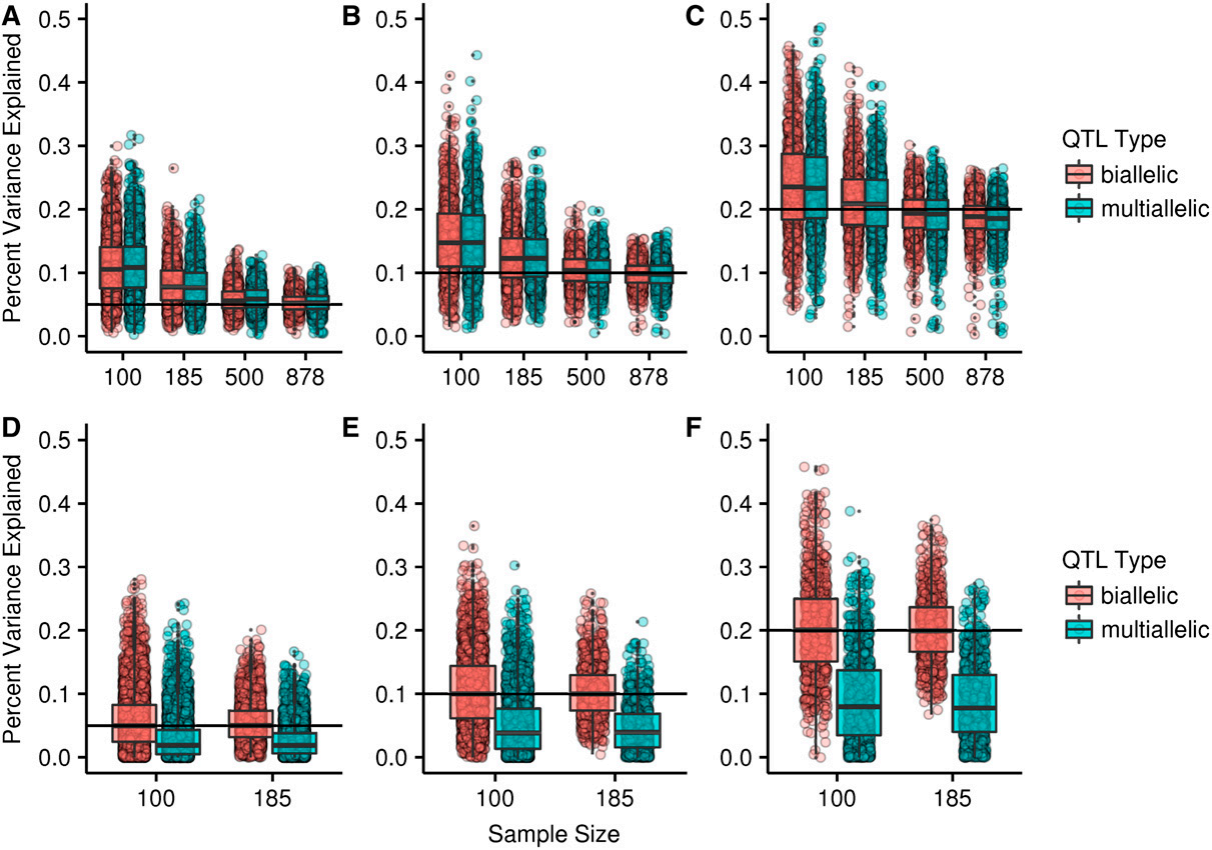
The percent variance explained by all simulated QTL at the true location in the DSPR (A–C) and the DGRP (D–F) for different sample sizes. The true percent variance explained by the QTL is shown by the solid line [(A and D) = 0.05, (B and E) = 0.1, and (C and F) = 0.2]. Each point corresponds to a single QTL. Boxplots are overlayed with the central line corresponding to the median and the box encompassing the first and third quartiles. DGRP, *Drosophila* Genetic Reference Panel; DSRP, *Drosophila* Synthetic Population Resource; QTL, quantitative trait loci.

**Figure 3:**
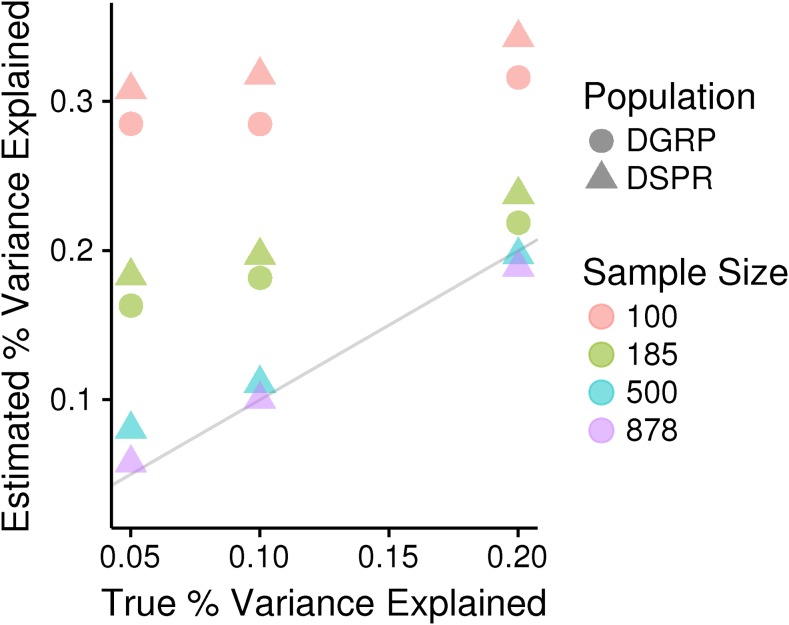
The average estimated percent variance explained by significant QTL *vs.* the true percent variance explained by those QTL for different sample sizes (colors) and mapping populations (symbols). Only simulated biallelic cases are shown. The 1:1 line is plotted in gray and represents where points should lie if estimated parameters match the true parameters. DGRP, *Drosophila* Genetic Reference Panel; DSRP, *Drosophila* Synthetic Population Resource; QTL, quantitative trait loci.

There is a small, but consistent difference between the mapping populations in the magnitude of the bias, with a larger bias in the DSPR. We hypothesize that the difference is due to the additional terms in the haplotype-based model and potential overfitting. The Beavis effect stems from conditioning on only significant QTL to estimate the PVE by QTL. If we instead estimated the PVE for all our simulated QTL at their true locations, regardless of whether they were found to be significant, the estimates are expected to be normally distributed and centered on the true magnitude. At the smallest sample sizes, both the DGRP and the DSPR deviate from this expectation, but the DSPR deviates much more severely (Figure 2). The DSPR model estimates an effect separately for each haplotype, but our simulated QTL are produced from fewer groups. For example, when the true effect is at a single SNP, just two groups are necessary. In addition, as sample size decreases, it becomes more likely that there will be little or in some cases no representation of some founder haplotypes at a given position. These haplotypes are nonetheless included in the model and this may contribute to an overfitting effect. However, we also note that the additional bias in the DSPR compared to the DGRP is quite small compared to the overall bias (∼2%; Figure 3).

Overall, the discovery of bias is not a new result, but it is worth noting that direct estimates of the PVE by QTL at low sample sizes are nearly wholly uninformative of the true effect, *i.e.*, the estimated PVE bears no relation to the true PVE (Figure 3). Despite this fact, the PVE by QTL are routinely reported for mapping studies with small sample sizes (*e.g.*, Aylor *et al.* 2011; Mackay *et al.* 2012; King *et al.* 2014a; Liu *et al.* 2016), and there is not an associated indicator of uncertainty such as a standard error to aid interpretation. Cross validation, which uses one portion of the data to identify significant QTL and the remaining data to estimate the PVE, has been shown to be an effective strategy to avoid bias in the estimation of the PVE attributable to QTL (Utz *et al.* 2000). However, this strategy is not routinely employed given that the full data set cannot be used to assess significance and there is a desire to maximize power. We advise explicitly acknowledging the lowest PVE that could be detected at a given sample size and the potential bias when cross validation is not employed.

### Significance thresholds and the Beavis effect

**Figure 4:**
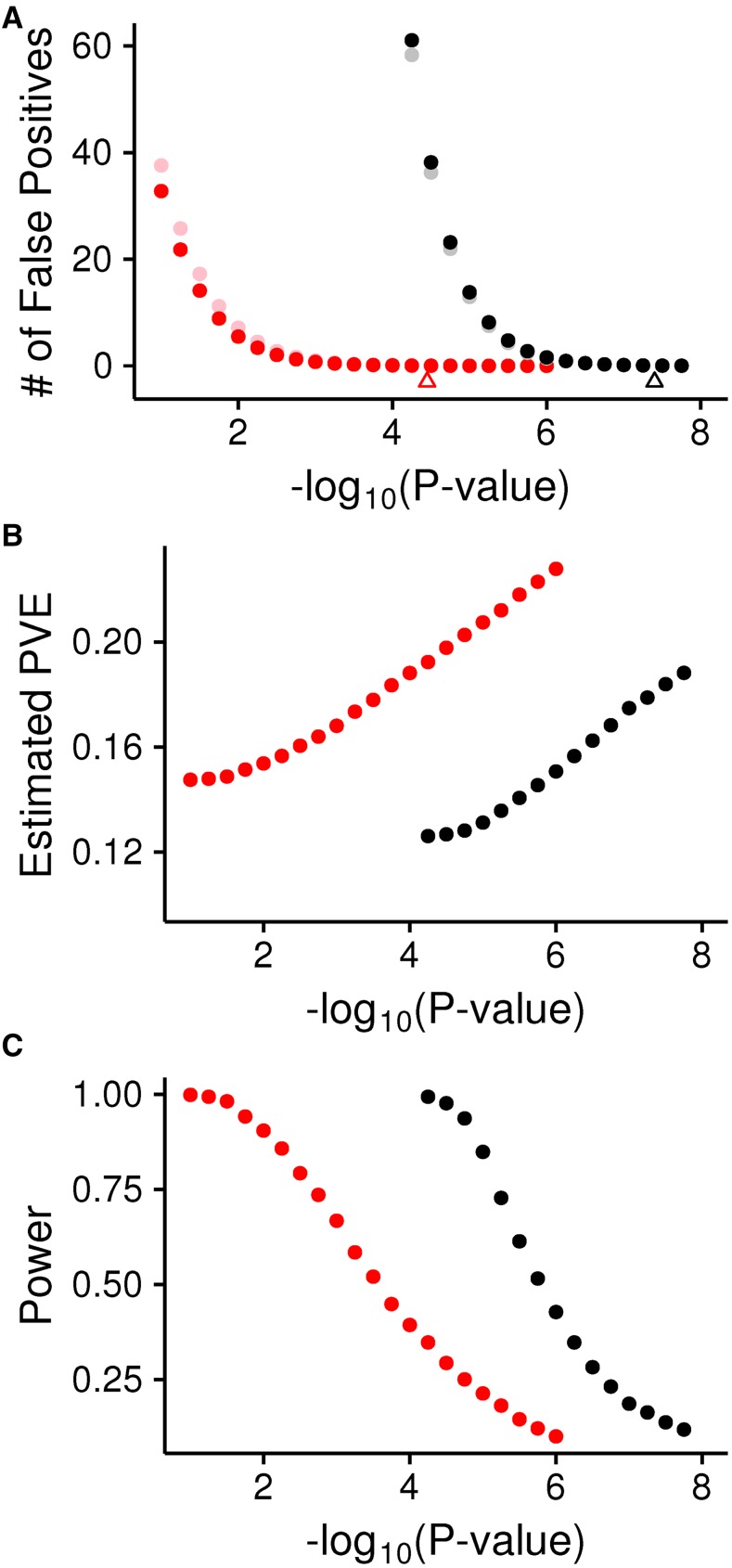
(A) The mean expected false positive rate for different *P*-value thresholds for the DSPR (red) and DGRP (black), with a sample size of 100 shown in light colors and the full panel size shown in dark (185 for DGRP and 878 for DSPR). (B) The mean estimated PVEexplained for different *P*-value thresholds for a true PVE of 10% and a sample size of 185. DSPR is in red and DGRP is in black. (C) Power for different *P*-value thresholds for a QTL with a PVE of 10% and a sample size of 185. DSPR is in red and DGRP is in black. DGRP, *Drosophila* Genetic Reference Panel; DSRP, *Drosophila* Synthetic Population Resource; PVE, percentage of variance explained; QTL, quantitative trait loci.

As described above, the Beavis effect results from choosing only the factors identified as significant, and thus choosing PVE at the tail of the overestimated side. Our results are thus dependent on the stringency of the significance threshold used. The above results controlled the family-wise error rate at 5%, which is a reliable but conservative strategy. Using more liberal criteria for significance will decrease the Beavis effect, but of course will also increase false positives at the same time. This trade-off is similar to the trade-off between the rate of false positives and false negatives. The Beavis effect can be reduced and power can be increased but only at the cost of increasing the false positive rate (Figure 4).

One of the major difficulties in assessing the potential true PVE from the estimated PVE, and how the bias might change with the significance threshold, is the lack of knowledge about the true distribution of effect sizes. For example, the probability that a given estimated PVE discovered in an experiment is mildly biased *vs.* severely biased is highly dependent on the underlying distribution of the true PVE of all causative variants. This distribution is typically assumed to be exponentially or γ distributed, with an abundance of low PVE loci and very few high PVE loci. As we have shown, the severity of the bias depends strongly on sample size, but it will also depend on this distribution of true PVE. For example, even if sample sizes are relatively high (*e.g.*, ∼1000), if the vast majority of causative variants contribute 1% or less to the phenotype, the resulting bias will generally still be quite severe, because power declines with decreasing PVE (Utz and Melchinger 1994; Beavis 1998; Xu 2003; Broman and Sen 2009).

### Prospects for validation of putative QTL

Within a mapping population, validation attempts typically involve attempting to map the same locus using a different experimental design, such as crossing many lines to create an outbred population, phenotyping this population, and resequencing a set of individuals from the tails of the distribution. Another method is crossing the lines to a standard line to perform phenotyping. When these methods are employed and there is little concordance between significant hits, a tempting conclusion is the presence of genetic background effects. Here, we simulated a QTL with identical parameters twice to determine the degree that one would expect replication in ideal circumstances. In this case, the likelihood of validating a true QTL could potentially be calculated by assessing power with our local region FWER. However, given a significant QTL in one study, the same genome-wide multiple testing criterion may not need to be applied if mapping is done locally. The power to validate a given QTL with our local region FWER threshold is shown in Table 2. It is also possible that individual loci may be more or less likely to replicate depending on other factors affecting power, such as frequency or the location on the chromosome. To account for this, we explicitly simulated new phenotypes for the loci deemed significant and performed mapping again. The resulting probability of validating QTL within populations is given in Table 1, though we note that in some parameter combinations with low power this probability is based on few loci. In most cases, the power to validate our previously identified loci is higher than considering all our simulated loci at the lower threshold, indicating that factors such as allele frequencies also influence the likelihood of validating a given locus. In many realistic parameter combinations (*e.g.*, a PVE of 5% and a sample size of a few hundred), the likelihood of validating a QTL is < 50% in both mapping populations. Furthermore, in many complex traits, the PVE of most causative loci are expected to be < 5%. Therefore, the failure of validation attempts within populations should not be taken as evidence for epistasis in the absence of additional evidence.

**Table 2:** Power (% QTL identified) to validate QTL in the DGRP and DSPR for different sample sizes and true PVE by the simulated QTL

		DGRP				DSPR			
		Biallelic		Multiallelic		Biallelic		Multiallelic	
True PVE	Sample Size	*N**^a^*	Mapped*^b^*	*N**^a^*	Mapped*^b^*	*N**^a^*	Mapped (%)*^b^*	*N**^a^*	Mapped (%)*^b^*
5	100	6000	356 (5.9%)	6000	299 (5.0%)	2000	248 (12.4)	2000	273 (13.7)
5	185	3000	252 (8.4%)	5000	338 (6.7%)	1000	247 (24.7)	1000	251 (25.1)
10	100	3000	282 (9.4%)	3000	216 (7.2%)	1000	299 (29.9)	1000	310 (31.0)
10	185	1000	319 (31.9%)	1000	163 (16.3%)	1000	617 (61.7)	1000	631 (63.1)
20	100	1000	424 (42.4%)	1000	218 (21.8%)	1000	69.5 (69.5)	1000	700 (70.0)
20	185	1000	940 (94.0%)	1000	638 (63.8%)	1000	972 (97.2)	1000	971 (97.1)
5	500	—	—	—	—	1000	781 (78.1)	1000	810 (81.0)
5	878	—	—	—	—	1000	984 (98.4)	1000	978 (97.8)
10	500	—	—	—	—	1000	992 (99.2)	1000	987 (98.7)
10	878	—	—	—	—	1000	997 (99.7)	1000	997 (99.7)
20	500	—	—	—	—	1000	998 (99.8)	1000	995 (99.5)
20	878	—	—	—	—	1000	999 (99.9)	1000	998 (99.8)

DGRP, *Drosophila* Genetic Reference Panel; DSRP, *Drosophila* Synthetic Population Resource; PVE, percentage of variance explained; QTL, quantitative trait loci.

a The number of simulations performed. Note that we simulated an increased number when power was low to generate enough mapped QTL to estimate the observed PVE accurately.

b The number simulations resulting in a significant, mapped QTL at the local region FWER threshold. The percentage (*i.e.*, power) is in parentheses.

**Figure 5:**
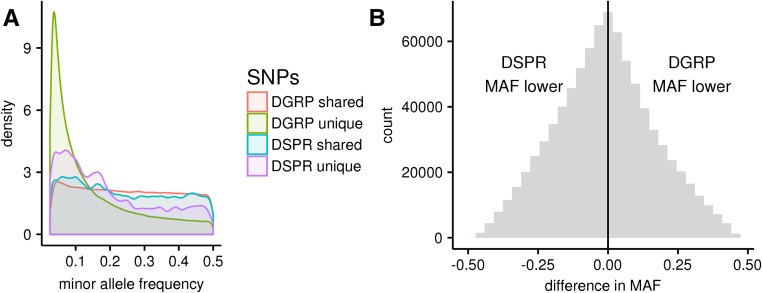
(A) Density plot of the frequencies of SNPs in the DGRP and DSPR. Different colors denote SNPs unique to each population and shared in the two populations. (B) Histogram of the differences in the MAF for the set of SNPs shared between the DGRP and the DSPR. The black vertical line denotes zero; to the left, the MAF in the DSPR is higher than in the DGRP; and to the right, the MAF is higher in the DGRP. DGRP, *Drosophila* Genetic Reference Panel; DSRP, *Drosophila* Synthetic Population Resource; MAF, minor allele frequency; QTL, quantitative trait loci; SNP, single nucleotide polymorphism.

Between populations, several additional factors besides a lower threshold will strongly influence the likelihood of validating a QTL in a second mapping population (*i.e.*, validating a DSPR hit in the DGRP or vice versa). First, a causative allele that is not segregating in the second population will obviously have no chance of being validated. In the DGRP and DSPR, there are a total of ∼2.5 million SNPs that passed our criteria, which excludes any SNP with a minor allele frequency of < 2.5% (see *Methods*). Of these, 60% are unique to the DGRP, 34% are shared between the two populations, and 7% are unique to the DSPR (Figure 5a). Thus, for biallelic QTL in these populations, only 34% of loci have a nonzero chance of being replicated. However, in the case of multiallelic QTL, where there are several SNPs within a causative QTL that affect the phenotype, the same precise SNPs do not necessarily need to be present for a QTL to replicate. There is a growing body of evidence in support of the hypothesis that mapped QTL can be multiallelic. For example, in the DSPR, there is evidence multiple alleles exist at causative QTL for large effect traits such as the *Adh* locus (King *et al.* 2012a) and large effect *cis* eQTLs (King *et al.* 2014b), a pattern that has also been found for human diseases [reviewed in McClellan and King (2010)] and is supported by theoretical models (Pritchard 2001; Thornton *et al.* 2013). Second, for an allele with a given additive effect, the allele frequency of the causative SNP will influence the PVE by the allele in the second population. In other words, if an allele with a given effect in one population is lower in frequency in the second population, it will explain a lower percentage of the variance. For the set of SNPs shared between the DGRP and the DSPR, the minor allele frequencies are correlated, but the relationship is not strong (*r* = 0.19, *P* < 0.001). Alleles are equally likely to be at increased frequency in the DSPR as in the DGRP, with an average difference of 14% (Figure 5b). Of course, this applies only to the set of shared SNPs in the two populations; there is a higher frequency of rare alleles in the DGRP but these alleles are unique to the DGRP. Finally, not only will the frequency of the causative locus vary, but the presence and frequency of all other causative variants will also vary, influencing the heritability of the trait and relative contributions of the loci. For a typical complex trait, the number of these variants is expected to be high. For these reasons, the expected PVE for a given locus in a second mapping population is essentially an unknown quantity. Thus, in the most general sense, the potential for validation is best informed by the power at our lower local region FWER for various PVE and sample sizes (Table 2).

However, for the set of shared SNPs in the DGRP and DSPR, it is useful to estimate the likelihood of replicating a locus between populations. For a subset of parameter combinations, we performed simulations to determine the power to identify a “hit” from the DGRP in the DSPR and vice versa (Table 3). Note that these simulations assume that the PVE of the locus is determined by the effect of the locus and its allele frequency and is not influenced by changes in the allele frequencies of other loci. For the same sample size, the power to validate between populations is similar, given that the SNP exists in both populations. The power to validate between populations for the full sample size in the DSPR is lower than the power to validate within populations (compare Table 2 and Table 3). Despite the fact that it is equally likely for an allele to increase or decrease in frequency, power is already high for the full sample size. Thus, SNPs at increased frequency in the DSPR compared to the DGRP (increasing the PVE) do not increase power substantially but SNPs at decreased frequency in the DSPR compared to the DGRP (decreasing the PVE) do reduce power, lowering overall power. Overall, as is generally the case in mapping studies, QTL would be expected to validate between populations when the SNP is common (increasing the likelihood it exists in another population), the PVE is high, and/or the sample size in the validation population is high.

**Table 3:** Power (% QTL identified) to validate QTL between populations

		Source = DGRP, Validation = DSPR		Source = DSPR, Validation = DGRP	
Source PVE	Validation Sample Size	Hits Shared Between Populations (%)*^a^*	Validated (%)*^b^*	Hits Shared Between Populations*^a^*	Validated*^b^*
5	185	27 (55.1)	6 (22.2)	837 (84.0%)	143 (17.1%)
5	878	27 (55.1)	17 (63.0)	—	—
10	185	77 (51.7)	35 (45.5)	1098 (84.5%)	464 (42.2%)
10	878	77 (51.7)	55 (71.4)	—	—

DGRP, *Drosophila* Genetic Reference Panel; DSRP, *Drosophila* Synthetic Population Resource; PVE, percentage of variance explained; FWER, family-wise error rate.

a The number of hits that also exist in the validation population with the percentage of hits that are shared in parentheses. These numbers vary according to the power in the initial simulation and the parameter combinations considered (see *Methods*).

b The number of shared hits also mapped in the validation population with the percentage (*i.e.*, power) in parentheses.

### Conclusions

Here, we have confirmed the behavior of the Beavis effect in two major modern *Drosophila* mapping populations, the multiparent DSPR and the GWAS-based DGRP. We have shown that the expected overestimation of the PVE by QTL is similar in the two populations and is strongly determined by sample size. Some of the severity of the Beavis effect stems from the requirement of using a high significance threshold that results from performing mapping at thousands to millions of positions. At one extreme, the false positive rate can be tightly controlled giving high confidence that a mapped QTL is a true positive; however, the Beavis effect will be most severe in this circumstance. Using relaxed significance thresholds may decrease the severity of the Beavis effect but will also increase the likelihood that any given QTL is a false positive. Approaches that use the entire set of SNPs to estimate the variance explained by all markers (*e.g.*, Yang *et al.* 2010) avoid both of these issues by not conditioning on specific loci as being significant. However, these methods are therefore unable to pinpoint specific loci as being potentially causative, instead describing the overall variance attributable to all genetic factors. Finally, we have shown that the power to validate a mapped QTL both within the same mapping population and between mapping populations is not necessarily high, even given the relaxed significance criteria that can be used for a focused scan. A lack of validation is generally the more likely scenario and should always be treated as an absence of evidence, not evidence of absence.

The core issues that we have discussed here—the PVE by QTL, power to detect QTL, and the prospects for validation of QTL—are relevant to any mapping population. One potential limitation to our simulations is that they only consider a single causative locus for each simulated phenotype. Our results are expected to be generalizable to any given mapped QTL, but we are not able to address issues that arise when mapping multiple QTL in a given experiment, including estimating the overall PVE explained by all QTL and the potential bias associated with this estimate. Qualitatively, our results in the DSPR should provide a rough guide for other MPPs (*e.g.*, McMullen *et al.* 2009; Aylor *et al.* 2011; Huang *et al.* 2011; Collaborative Cross Consortium 2012; Cubillos *et al.* 2013) employing haplotype-based mapping. In addition, while power is quite different in the DSPR and DGRP, the bias in PVE in the two resources is quite similar for similar sample sizes. This result suggests that our results, and those found previously (Utz and Melchinger 1994; Beavis 1998; Xu 2003; Broman and Sen 2009), for the bias in PVE may apply very broadly across mapping designs as well, though this issue deserves a more thorough investigation of additional mapping panels and designs in various species.

## Supplementary Material

Supplemental material is available online at www.g3journal.org/lookup/suppl/doi:10.1534/g3.117.041426/-/DC1.
