# Supplementary material for: The Beavis Effect in Next-Generation Mapping Panels in Drosophila melanogaster
Source: G3 (Bethesda). 2017 Jun 5;7(6):1643–52. doi: 10.1534/g3.117.041426 (PMC5473746; doi:10.1534/g3.117.041426)
Supplement: Supplementary file 1 [file 1643File001.pdf]

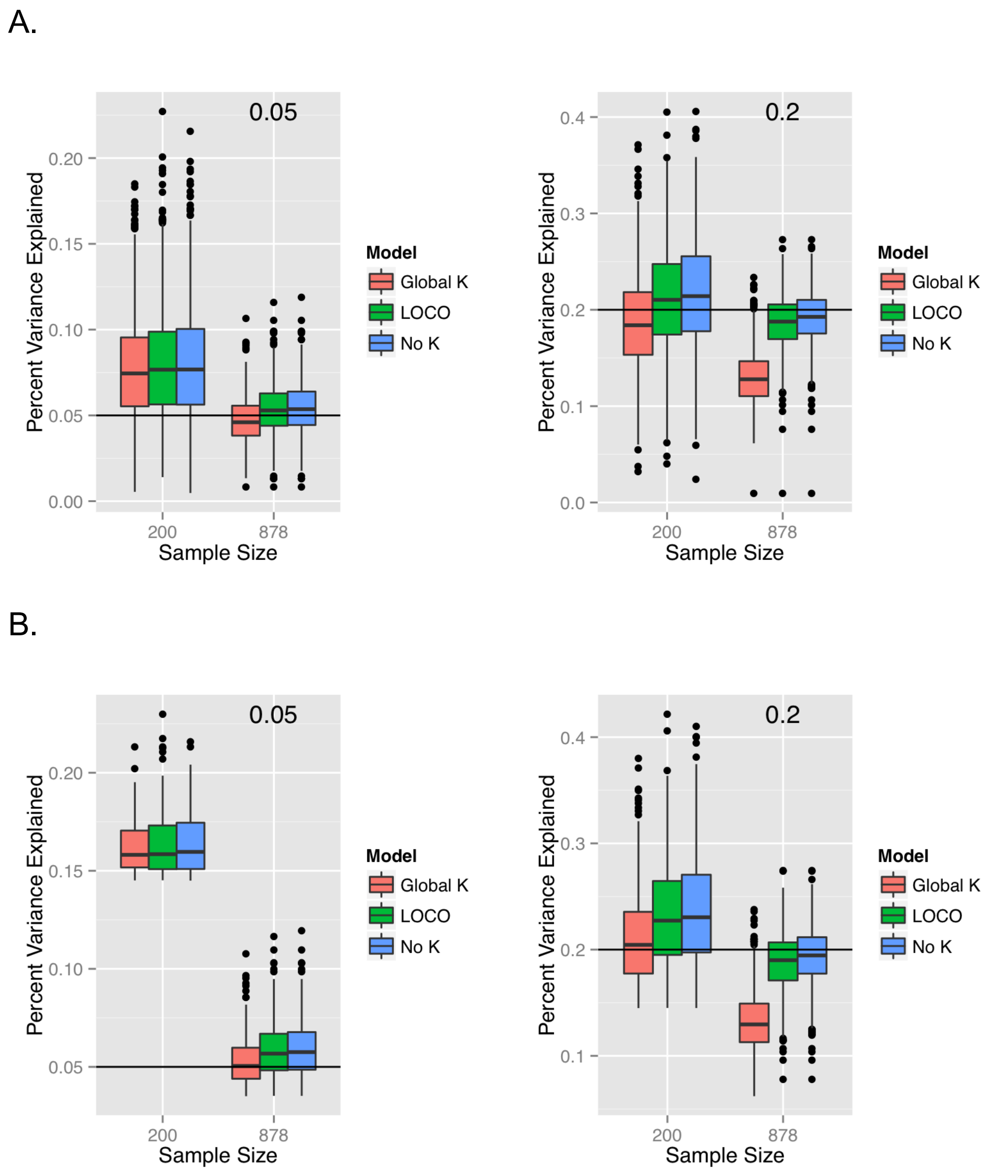

Figure S1: A. PVE estimates for all simulated QTL in the DSPR regardless of whether the QTL was deemed significant. B. PVE estimates for simulated QTL in the DSPR that were significant at an FWER of 5%. Different colors denote different mapping methods. Global K indicates a single overall kinship matrix correction, LOCO is the leave-one-chromosome-out method of kinship correction, and no K is no kinship correction.

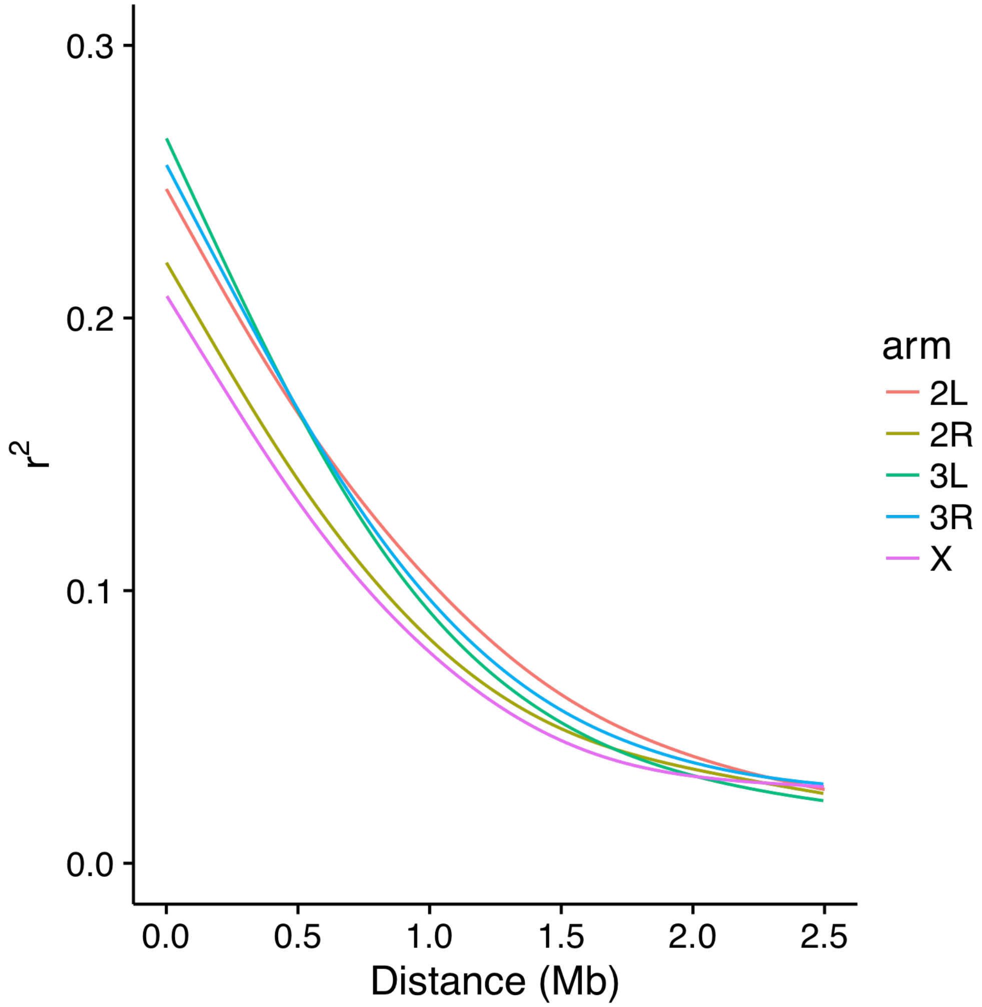

Figure S2: Decay of linkage disequilibirum (r^2) with distance for each chromosome arm (different colors).
